# Neurophysiological prediction of competitive orientation via EEG during outcome evaluation in gamified learning

**DOI:** 10.1038/s41539-026-00420-y

**Published:** 2026-04-06

**Authors:** Hiroki Watanabe, Ayato Tabata, Chikara Ishii, Yasushi Naruse

**Affiliations:** Center for Information and Neural Networks, Advanced ICT Research Institute, National Institute of Information and Communications Technology, and Osaka University, 651-2492 Kobe, Japan

**Keywords:** Neuroscience, Psychology, Psychology

## Abstract

Gamification often employs competition-based mechanics to enhance motivation; however, their effectiveness varies depending on an individual’s competitive orientation. This study proposes a method to assess competitive orientation using EEG data in a gamified learning environment. Twenty-eight participants completed a gamified mental arithmetic task under two conditions: competing against an opponent (competitive condition) and attempting to surpass their best score (self-referential condition). Event-related potentials (ERPs) in response to outcome feedback—P300, feedback-related negativity, and reward positivity (RewP)—were analyzed. Results showed that RewP amplitude in the competitive condition was significantly correlated with competitive orientation scores. P300 amplitude was associated with the scores regardless of condition. Furthermore, a support vector regression model trained on ERP and behavioral features from the competitive condition predicted competitive orientation scores (*r* = 0.468). These findings suggest that ERP responses provide objective information for estimating competitive orientation, highlighting a neurophysiological approach to personalized gamification design.

## Introduction

Advancements in digital technology have significantly transformed the education sector. The widespread use of the internet and digital learning devices, such as laptops and tablets, has established an environment where students can actively engage in learning through online education and interactive tools^1^. For instance, the advancement of data mining techniques for analyzing big educational data accumulated in learning management systems has enabled personalized learning support^2^. Research has demonstrated that multimedia materials that combine words and pictures can improve learning and comprehension^3^. Therefore, the use of digital technology facilitates better student comprehension and more efficient instruction, contributing to the overall improvement of educational quality.

Among various educational digital technologies, gamification has appeared as a promising approach to improve learners’ engagement and motivation. Gamification, which is “the use of game design elements in non-game contexts ”^4^, has gained significant attention in the field of education^5^. A review of recent studies indicates that integrating game elements into learning environments can improve engagement and motivation when implemented with valid context and appropriate strategies^6^. Typical game elements used in the field of education include badges and points for achievements, leaderboards for rankings, and levels that learners progress through. These elements aim to create a rewarding learning experience. Among the game elements, the effects of competition have been studied as a mechanism in gamification within educational settings^7^, including team competition^8,9^, leaderboards^10,11^, and surrogate competition, where educational agents act as intermediaries between students in competitive learning environments^12^. Competition is one of the motivational factors in online games^13^. It supports learners’ need for competence^14^, which is a fundamental psychological need for intrinsic motivation based on self-determination theory (SDT)^15^. However, research indicates that the effectiveness of competitive elements differs according to individual differences in competitive orientation—defined as individual differences in the extent to which individuals enjoy competition. For instance, Song et al. (2013) revealed that users with a high competitive orientation benefit from competition-based game elements, whereas those with a low competitive orientation experience detrimental effects^16^. Further, within the framework of achievement goal theory, similar moderating effects of individual differences have been observed. Achievement goal theory distinguishes between performance goals, which are grounded in normative standards and focus on outperforming others, and mastery goals, which are based on self-referential standards and emphasize learning, self-improvement, and personal progress^17^. Within this framework, prior research indicates learners with a high achievement orientation—a general tendency to strive for excellence by seeking challenge and outperforming others—tend to develop greater interest in a math problem-solving task when exposed to performance goals. In contrast, learners with a low achievement orientation are more likely to benefit from mastery goals^18^. These findings suggest that the effectiveness of competitive elements is contingent on their congruence with learners’ individual differences related to competitiveness.

Thus far, questionnaires have been primarily employed as a method for assessing an individual’s competitive orientation. The BrainHex questionnaire^19^ was developed to assess individual preferences in gaming experiences and categorize players into different player types. One of these player types, “conqueror,” is strongly associated with competitive orientation and is defined as “players fitting the conqueror archetype enjoy defeating impossibly difficult foes, struggling until they achieve victory, and beating other players^19^.” In this questionnaire, subjective assessment results are scored from −10 to 20 for each type, thereby evaluating how well an individual fits into each category. The BrainHex questionnaire has been applied for player type categorization in gamification systems that personalize the selection of game elements based on individual differences^20^. Therefore, the quantification of competitive orientation that is used in this questionnaire exhibits a certain level of usefulness. However, self-report methods such as questionnaire responses are susceptible to various response biases, including socially desirable responding; acquiescent responding (i.e., the tendency to agree with statements regardless of their content); extreme responding (i.e., the tendency to select extreme response options, such as “1” or “7” on a 7-point rating scale); pattern responding, in which participants mark responses in a fixed physical pattern, such as selecting the same response option (e.g., always choosing “3” on a 7-point rating scale); and random responding, in which answers are given indiscriminately^21^. Such biases may compromise response validity, making it difficult to accurately identify learners’ competitive orientation. Accordingly, this limitation suggests the need for an objective assessment approach that evaluates learners’ competitive orientation based on their actual state during the learning process.

One promising objective approach to assessing learners’ competitive orientation is to evaluate their psychophysiological responses to competitive elements in gamified digital learning environments, particularly in situations where the goal is to outperform others. Competitive orientation may impact the extent of one’s motivation and engagement in pursuing competitive goals. Therefore, individual differences in competitive orientation are expected to be inferred from changes in psychophysiological responses associated with psychological states that are involved in goal pursuit and achievement. Among these responses, electroencephalography (EEG) has attracted attention as a practical and feasible method for assessing psychological states, particularly in real-world settings such as educational environments. Recent technological advancements have resulted in the development of compact and wearable EEG devices, enabling non-invasive brain activity measurement in real-life situations^22,23^. Further, the use of active dry electrodes, which do not require conductive gel, has significantly improved the comfort and usability of EEG measurements. These innovations enable EEG to be seamlessly integrated into digital learning materials without disrupting the learning process.

In addition to its practical use, previous studies have indicated that event-related potentials (ERPs), which are observed during learning tasks, are associated with psychological states involved in goal pursuit and achievement. In our previous study, we demonstrated that the strength of learners’ motivation toward goal achievement could be assessed through these neural responses^24^. In the experiment, participants performed a task in which they received feedback that indicated whether their answer was correct or not for each problem in the mental arithmetic task. They were provided individual goal scores tailored to their abilities. The results revealed a positive correlation between the amplitude of the P300 in response to incorrect feedback and the learners’ subjective motivation to achieve the goal score. P300 is a positive ERP component that is observed in the parietal region, approximately 300 ms after stimulus presentation, and is known to reflect attentional allocation and the motivational significance of stimuli^25,26^. Therefore, learners with higher motivation to achieve the task goal paid greater attention to incorrect answers, which are essential sources of learning-related information. Taken together, under competitive goal structures that emphasize outperforming opponents, learners’ motivation toward goal achievement varies according to their level of competitive orientation, which in turn can lead to changes in attentional allocation. Therefore, measuring ERPs that reflect such changes in attentional allocation may provide a useful basis for estimating individual differences in competitive orientation.

In addition to P300, feedback-related negativity (FRN) and reward positivity (RewP) have been associated with psychological states involved in goal pursuit and achievement. FRN and RewP are ERP components that are observed in the frontocentral region approximately 250 ms after feedback presentation and are considered to reflect negative and positive reward prediction errors, respectively^27–30^. FRN is typically generated in response to outcomes worse than expected (e.g., incorrect answers). Concurrently, RewP appears in response to outcomes that are better than expected (e.g., correct answers or rewards)^28,29^. Psychological factors, such as motivation during goal pursuit, may modulate sensitivity to performance feedback and its evaluative value, thereby causing changes in FRN and RewP amplitudes^31^. These reward prediction error-related neural responses may reflect individual differences in sensitivity to feedback and value assessment, particularly when pursuing the goal of winning in competitive contexts based on the competitive orientation level. Thus, FRN and RewP may contribute to estimating competitive orientation. However, variations of these neural responses based on individual differences in competitive orientation, particularly in contexts that align with one’s orientation, such as gamified learning environments in which learners aim to outperform an opponent, remain unclear. Therefore, the aim of this study is to examine the association between these neural responses during the pursuit of competitive goals and individual differences in competitive orientation, as well as to clarify the contribution of these neural responses to estimating learners’ competitive orientation.

**Fig. 1:**
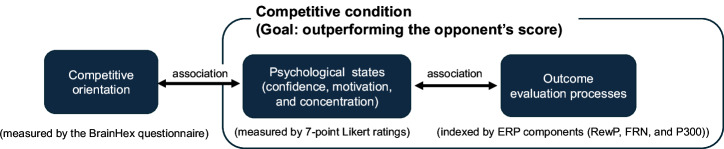
Conceptual framework of the present study illustrating the hypothesized associations among competitive orientation, psychological states, and outcome evaluation processes within the competitive condition. The figure depicts the hypothesized associations between competitive orientation (measured by the BrainHex questionnaire), psychological states including confidence, motivation, and concentration (measured by 7-point Likert ratings), and outcome evaluation processes indexed by event-related potential (ERP) components, namely reward positivity (RewP), feedback-related negativity (FRN), and P300, under the competitive condition in which participants aimed to outperform an opponent’s score.

To this end, this study has two primary objectives. The first objective is to determine whether measurements of ERPs objectively reflect competitive orientation by analyzing their association with learners’ competitive orientation scores (COS), as measured by the BrainHex questionnaire^19^, in a gamified learning environment. Specifically, this study investigates whether P300, RewP, and FRN correlate with competitive orientation in a competitive environment but not in a self-referential environment that emphasizes achieving one’s personal best score. Individual differences in competitive orientation are expected to play a role primarily in competitive contexts, but not in self-referential contexts, in which performance evaluation is based on one’s own standards rather than interpersonal comparison. This contrast is conceptually aligned with the experimental manipulation used by Barron and Harackiewicz (2001) within the framework of achievement goal theory^18^. Thus, an EEG measurement experiment was conducted, where participants engaged in a learning task under competitive and self-referential conditions. In the competitive condition, each pair of participants competed to achieve the goal of obtaining higher scores than their opponent. To improve the realism of the competition, participants were paired into groups of two and performed the task as direct opponents. In the self-referential condition, participants attempted to surpass their own predetermined best scores. In both conditions, the goal was set according to each participant’s highest score, ensuring that it was equally achievable. This score determined whether the goal was achieved, thereby creating identical conditions aside from the instructions. The learning task consisted of a gamified mental arithmetic exercise presented via a graphical user interface (GUI) on a laptop. Mental arithmetic was chosen because, in addition to its use in previous research^24^, it allowed fine-grained control of task difficulty while minimizing the influence of prior domain-specific knowledge. In addition, the probability of correct responses due to random guessing is substantially lower than in forced-choice tasks. Participants were instructed to aim to improve their task performance across a total of four sessions. Each session consisted of 48 addition problems categorized into three difficulty levels (16 problems per level), which were individually selected from six predefined levels based on each participant’s performance in the practice session. The same set of difficulty levels was used across both the competitive and self-referential conditions to ensure consistency between conditions. During the task, P300, RewP, and FRN responses to outcome feedback were recorded in both conditions. Participants received visual success/failure feedback after each session and rated their confidence (before the session) as well as motivation and concentration (after the session) using 7-point Likert scales (subjective ratings). In this study, confidence in achieving the goal was rated as an anticipatory motivational state reflecting learners’ expectancy of success and perceived competence before engaging in a goal-directed learning task. Hence, we hypothesize that these responses are associated with COS, as measured by the BrainHex questionnaire, and that this association emerges only under competitive goal conditions, but not under the self-referential condition. Figure 1 presents a conceptual framework of the present study illustrating the hypothesized associations among competitive orientation, psychological states, and outcome evaluation processes within the competitive condition.

The second objective is to examine whether ERP features contribute to the prediction of competitive orientation. To this end, ERP features together with behavioral and covariate features were utilized to construct a support vector regression (SVR) model with a linear kernel for predicting COS. In SVR, the *ϵ* -insensitive loss function does not penalize prediction errors whose absolute values are within *ϵ* during training, making the model less sensitive to small fluctuations in the data, such as measurement noise commonly observed in EEG signals. Consequently, this property may help reduce overfitting to noise and improve generalization performance. In addition, the regularization parameter helps control model complexity. Further, a linear model is beneficial for interpretability, as it indicates which features contribute to the prediction, making it suitable for assessing the effectiveness of ERP measurements.

## Results

### Effects of COS congruence with competitive elements on psychological factors and task performance

To investigate our hypothesis, first, we assessed the foundational assumption that congruence (or incongruence) between COS and competitive game elements affects psychological factors, i.e., subjective ratings, using a linear mixed-effects model (LME). The model included COS, condition (competitive or self-referential), and their interaction as well as the session and its interaction with the condition set as fixed effects. Task score was also included as a covariate to control for performance-related effects on subjective responses.

**Fig. 2:**
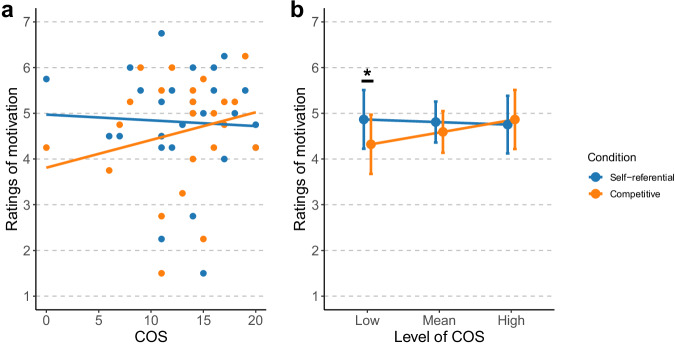
Results of the subjective rating analyses. **a** Association between competitive orientation score (COS) and subjective ratings of motivation. Lines illustrate predicted values based on coefficients from the fitted models. Dots indicate the mean for each participant. **b** Estimated marginal means of subjective ratings of motivation by condition at different levels of COS. Points represent model-estimated marginal means at low (− 1 SD), mean, and high (+ 1 SD) levels of COS. Error bars indicate 95% confidence intervals. An asterisk indicates a significant between-condition difference at the low COS level (*p* < 0.05).

Table 1 summarizes the LME analyses on the subjective ratings. Importantly, for motivation, we revealed a significant interaction between COS and condition (*F* = 5.35, *p* = 0.022), indicating that the effect of COS on motivation was modulated based on whether participants were in a competitive context or not. The post-hoc tests revealed that motivation slightly decreased in the self-referential condition as COS increased (*β* = − 0.06, *t* = − 0.26, *p* = 0.800), whereas it increased in the competitive condition (*β* = 0.27, *t* = 1.21, *p* = 0.237) (Fig. 2a). However, it did not reach statistical significance. In addition, we examined the simple effects of condition at different levels of COS. At low COS (− 1 SD), motivation was significantly lower in the competitive condition than in the self-referential condition (*β* = 0.54, *t* = 2.73, *p* = 0.021). At the mean level of COS and at high COS (+ 1 SD), this difference was attenuated and did not reach statistical significance (mean COS: *β* = 0.22, *t* = 1.51, *p* = 0.268; high COS: *β* = − 0.11, *t* = − 0.55, *p* = 0.584) (Fig. 2b).

**Table 1:** Results of linear mixed-effects models examining the effects of competitive orientation and condition on subjective ratings of motivation, concentration, and confidence

Fixed effects	Estimate (*β*)	SE	*F*	*p*	Significance
**Motivation**					
Intercept	4.70	0.21	–	–	–
COS	0.11	0.21	0.26	0.615	–
Condition	0.11	0.07	2.27	0.134	–
Session	−0.16	0.07	5.02	0.026	*
(Score)	0.36	0.13	7.94	0.005	*
COS × Condition	−0.16	0.07	5.35	0.022	*
Condition × Session	−0.10	0.07	2.14	0.145	–
**Concentration**					
Intercept	4.78	0.21	–	–	–
COS	0.04	0.21	0.04	0.848	–
Condition	0.07	0.08	0.83	0.364	–
Session	0.10	0.08	1.60	0.208	–
(Score)	0.39	0.14	8.00	0.005	*
COS × Condition	−0.14	0.08	3.07	0.082	–
Condition × Session	0.09	0.08	1.38	0.241	–
**Confidence**					
Intercept	3.57	0.22	–	–	–
COS	0.41	0.22	3.41	0.077	–
Condition	−0.05	0.08	0.42	0.516	–
Session	−0.21	0.08	6.57	0.011	*
(Score)	0.19	0.14	1.89	0.172	–
COS × Condition	−0.08	0.08	0.92	0.339	–
Condition × Session	−0.14	0.08	3.21	0.075	–

*Notes*. *SE* Standard error. *p < 0.05.

Covariates are in parentheses.

Regarding other significant effects, both motivation and confidence decreased as the session progressed (motivation: *β* = − 0.16, *F* = 5.02, *p* = 0.026; confidence: *β* = − 0.21, *F* = 6.57, *p* = 0.011). This decline may be caused by task-related fatigue or the increasing difficulty of the target score over time. In contrast, motivation and concentration ratings increased as task scores raised (motivation: *β* = 0.36, *F* = 7.94, *p* = 0.005; concentration: *β* = 0.39, *F* = 8.00, *p* = 0.005), indicating a positive association between task performance and psychological factors associated with motivation.

**Fig. 3:**
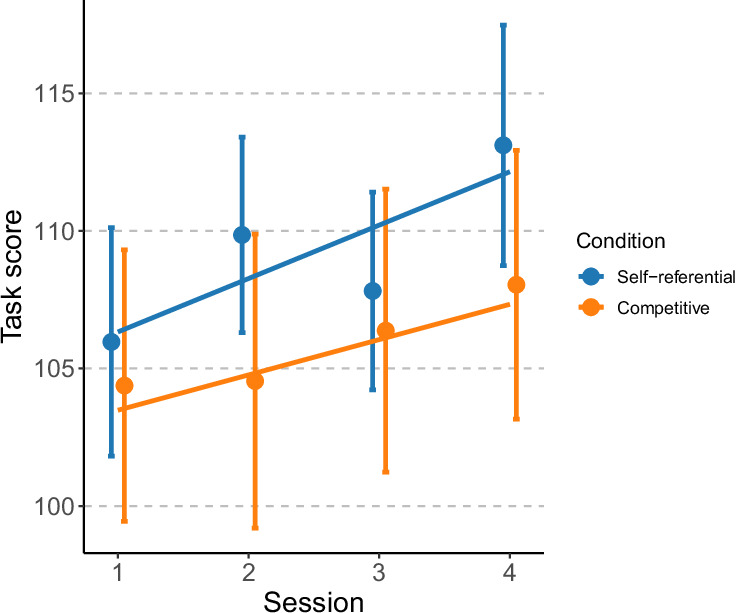
Task scores for each session and condition under self-referential and competitive conditions. Lines represent predicted values according to the coefficients from the fitted models. Points indicate the mean task score across participants for each session and condition, and error bars indicate the standard error of the mean across participants.

Subsequently, we investigated their effects on task performance, i.e., task score. The model included COS, condition, and their interaction as well as the session and its interaction with the condition set as fixed effects. Table 2 summarizes the LME analyses on task scores. A significant main effect of the session was found (*β* = 1.81, *F* = 5.07, *p* = 0.026), indicating that participants’ task performance improved over the sessions (Fig. 3). Further, we revealed a significant main effect of condition (*F* = 5.43, *p* = 0.021). The task score during the self-referential condition was superior to that during the competitive condition, even though analyses of the subjective ratings did not reveal a main effect of condition. The interaction between COS and condition was not significant (*F* = 0.33, *p* = 0.564).

**Table 2:** Results of a linear mixed-effects model examining the effects of competitive orientation and condition on task performance

Fixed effects	Estimate (*β*)	SE	*F*	*p*	Significance
**Task score**					
Intercept	107.32	3.79	–	–	–
COS	−3.48	3.81	0.84	0.369	–
Condition	1.92	0.82	5.43	0.021	*
Session	1.81	0.80	5.07	0.026	*
COS × Condition	0.48	0.82	0.33	0.564	–
Condition × Session	0.37	0.80	0.21	0.647	–

*Notes*. *SE* Standard error. *p < 0.05.

### Effects of COS congruence with competitive elements on ERP components

We examined the association between ERP mean amplitudes and COS using LMEs, including condition (competitive vs. self-referential) and their interaction. Separate models were fitted for each ERP component. Single-trial mean amplitudes of the RewP, FRN, and P300 elicited by outcome feedback for each arithmetic problem were used as response variables in a single-trial analysis approach^32^. Fixed effects included COS, condition, and their interaction, as well as session and its interaction with condition. The difficulty level of each addition problem and local frequency were included as covariates. Local frequency captured expectancy-related effects and was defined as the number of consecutive incorrect trials preceding a correct response for the RewP, and the number of consecutive correct trials preceding an incorrect response for the FRN and P300.

**Fig. 4:**
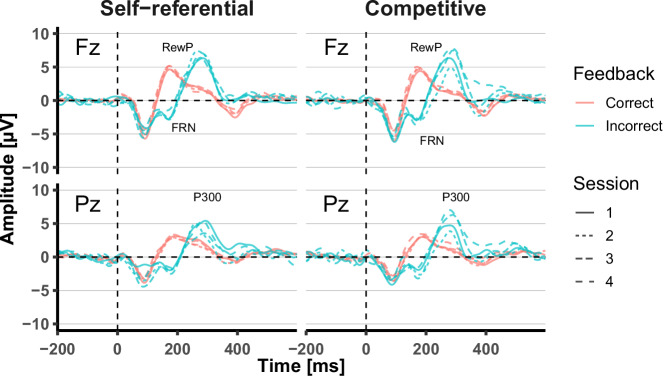
Grand-averaged event-related potentials (ERPs) for each condition, session, feedback type, and electrode site across participants. Grand-averaged ERP waveforms for the self-referential and competitive conditions are shown at electrodes Fz and Pz, separately for correct and incorrect feedback in each session. The reward positivity (RewP), feedback-related negativity (FRN), and P300 components are indicated.

Figure 4 presents the grand-averaged ERPs time-locked to the feedback onset. RewP, FRN, and P300 are observed at approximately 200 ms and 300 ms at Fz and Pz, respectively. Table 3 summarizes the LME analyses.

**Table 3:** Results of linear mixed-effects models examining the effects of competitive orientation and condition on event-related potential (ERP) amplitudes

Fixed effects	Estimate (*β*)	SE	*F*	*p*	Significance
**Reward Positivity (RewP)**					
Intercept	3.79	0.32	–	–	–
COS	−0.62	0.33	3.61	0.069	–
Condition	0.13	0.07	3.85	0.0498	*
Session	0.26	0.07	15.66	<0.001	*
(Difficulty)	0.57	0.11	29.58	<0.001	*
(Local frequency)	0.05	0.07	0.63	0.428	–
COS × Condition	0.13	0.07	4.09	0.043	*
Condition × Session	−0.06	0.07	0.77	0.382	–
**Feedback-Related Negativity (FRN)**					
Intercept	−1.80	0.52	–	–	–
COS	0.29	0.53	0.31	0.585	–
Condition	0.26	0.20	1.66	0.198	–
Session	0.15	0.20	0.60	0.440	–
(Difficulty)	0.84	0.27	9.31	0.002	*
(Local frequency)	−0.64	0.21	9.45	0.002	*
COS × Condition	0.10	0.20	0.25	0.615	–
Condition × Session	−0.08	0.20	0.17	0.679	–
**P300**					
Intercept	3.64	0.30	–	–	–
COS	−1.05	0.30	11.92	0.003	*
Condition	−0.20	0.19	1.19	0.276	–
Session	0.07	0.18	0.15	0.703	–
(Difficulty)	0.10	0.23	0.18	0.674	–
(Local frequency)	0.67	0.19	12.58	<0.001	*
COS × Condition	0.01	0.19	0.00	0.955	–
Condition × Session	−0.47	0.18	6.75	0.001	*

*Notes*. *SE* standard error. *p < 0.05. Covariates are in parentheses.

**Fig. 5:**
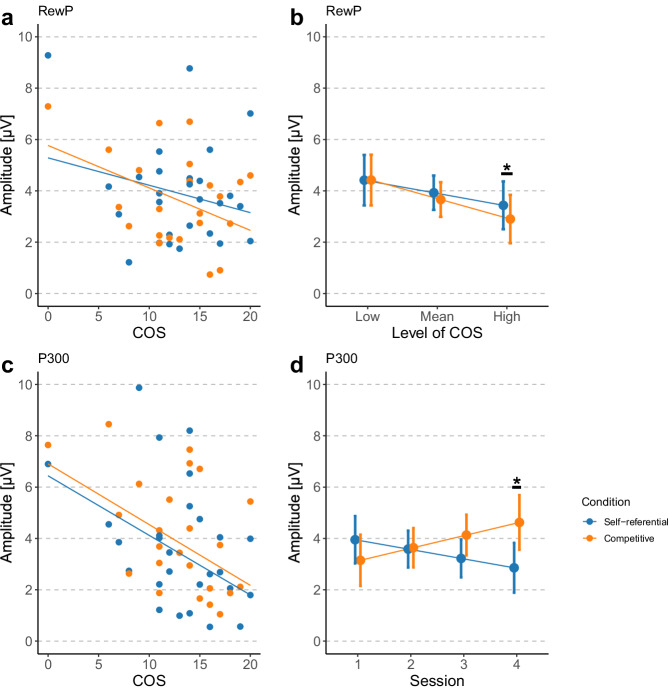
Results of the event-related potential (ERP) analyses. **a** Association between competitive orientation score (COS) and reward positivity (RewP) amplitudes. Lines illustrate predicted values based on coefficients from the fitted models. Dots indicate the mean for each participant. **b** Estimated marginal means of RewP amplitudes by condition at different levels of COS. Points represent model-estimated marginal means at low (− 1 SD), mean, and high (+1 SD) levels of COS. Error bars indicate 95% confidence intervals. An asterisk indicates a significant between-condition difference at the high COS level (*p* < 0.05). **c** Association between COS and P300 amplitudes. Lines illustrate predicted values based on coefficients from the fitted models. Dots indicate the mean for each participant. **d** Estimated marginal means of P300 amplitudes by condition for each session. Points represent model-estimated marginal means for each session. Error bars indicate 95% confidence intervals. An asterisk indicates a significant between-condition difference in Session 4 (*p* < 0.05).

For the RewP component, a significant main effect of condition (*F* = 3.85, *p* = 0.0498) and a significant interaction was observed between COS and condition for RewP amplitude (*F* = 4.09, *p* = 0.043), indicating that the association between competitive orientation and reward-related neural activity depended on the competitive context. Post-hoc analyses revealed that individuals with higher COS demonstrated reduced RewP amplitudes in the competitive condition (*β* = − 0.76, *t* = − 2.26, *p* = 0.032). Concurrently, this association was not statistically significant in the self-referential condition (*β* = − 0.49, *t* = − 1.47, *p* = 0.154) (Fig. 5a). In addition, simple effects analyses examining the effect of condition at different levels of COS revealed that, at high COS (+ 1 SD), mean RewP amplitudes were significantly decreased in the competitive condition compared with the self-referential condition (*β* = 0.53, *t* = 2.80, *p* = 0.017). In contrast, at low and mean levels of COS (− 1 SD), mean RewP amplitudes did not differ significantly between conditions (low COS: *β* = − 0.01, *t* = − 0.03, *p* = 0.978; mean COS: *β* = 0.26, *t* = 1.96, *p* = 0.100) (Fig. 5b). Further, the significant main effects of session (*β* = 0.26, *F* = 15.66, *p* < 0.001) and difficulty (*β* = 0.57, *F* = 29.58, *p* < 0.001) were found.

For the FRN component, significant main effects were observed for difficulty (*β* = 0.84, *F* = 9.31, *p* = 0.002) and local frequency covariates (*β* = − 0.64, *F* = 9.45, *p* = 0.002) but not for condition or COS.

Regarding the P300 component, a significant main effect of COS was observed (*β* = − 1.05, *F* = 11.92, *p* = 0.003), indicating that individuals with higher COS consistently demonstrated reduced P300 amplitudes across both conditions (Fig. 5c). In addition, a significant interaction between condition and session was observed (*F* = 6.75, *p* = 0.001). Post hoc analyses revealed that P300 amplitudes increased significantly across sessions in the competitive condition (*β* = 0.55, *t* = 1.98, *p* = 0.048), whereas no significant change was observed in the self-referential condition (*β* = − 0.40, *t* = − 1.65, *p* = 0.099) (Fig. 5d). Simple effects analyses examining the effect of condition at each session revealed no significant differences between conditions in Sessions 1–3 (Session 1: *β* = 0.80, *t* = 1.33, *p* = 0.366; Session 2: *β* = − 0.05, *t* = − 0.14, *p* = 0.892; Session 3: *β* = − 0.91, *t* = − 2.18, *p* = 0.089). In contrast, a significant difference was observed in Session 4 (*β* = − 1.77, *t* = − 2.77, *p* = 0.023). Further, local frequency (*β* = 0.67, *F* = 12.58, *p* < 0.001) was significantly associated with amplitudes.

### Prediction of COS

The features used for prediction included ERP measures (mean amplitudes of RewP to correct trials, FRN to incorrect trials, and P300 to incorrect trials), as well as task scores in the competitive condition. To account for potential confounding factors that may affect ERP amplitudes, three covariates were included as predictive features: the difficulty level of incorrect trials, the difficulty level of correct trials, and the local frequency of incorrect trials. ERP features, as well as the difficulty levels of incorrect and correct trials and the local frequency of incorrect trials, were originally defined at the single-trial level. Therefore, these variables were first averaged within each session and then averaged across sessions for each participant. In contrast, task scores represent a session-level outcome and were averaged across sessions only. Local frequency based on correct trials was not included because it conveys largely overlapping information with the incorrect-trial-based measure and would increase redundancy in the feature set. Hence, the final feature set comprised seven features. Models were evaluated using leave-one-out cross-validation (LOOCV). To examine the contribution of each feature to the prediction, the mean absolute values of the coefficients assigned to each feature across all trained models were interpreted as feature importance scores.

**Fig. 6:**
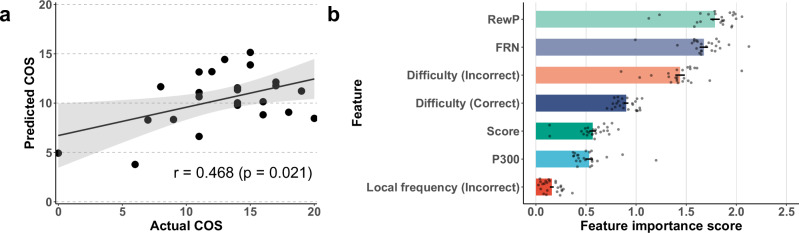
Results of the competitive orientation score (COS) prediction. **a** Comparison between predicted and actual COSs. **b** Average feature importance scores across all models trained during the leave-one-out cross-validation process. Dots indicate the coefficients from each model. Horizontal lines denote the standard error of the mean.

The regression analysis revealed that SVR effectively predicted individuals’ COSs (Fig. 6a). The model achieved a mean absolute error (MAE) of 3.68 and a significant *r* of 0.468 (*p* = 0.021) between the actual and predicted scores by the model. Figure 6b shows the feature importance scores across all models trained during the LOOCV. The features most contributing to the regression were RewP amplitudes, followed by FRN, indicating the effectiveness of ERP features for prediction.

We additionally evaluated other linear regression models (ridge, lasso, and elastic net) using the same feature set and cross-validation procedure as in the SVR analysis. None of these alternative models outperformed SVR in terms of predictive performance, as measured by Pearson’s *r* (Supplementary Table 1). Furthermore, we evaluated a reduced feature set that included only the two ERP features with the highest contributions (RewP and FRN), as identified by the feature importance scores. This reduced model showed lower predictive performance than the full feature set (Supplementary Table 2).

## Discussion

The present study primarily aimed to investigate whether ERPs—specifically P300, RewP, and FRN in response to outcome feedback—objectively reflect competitive orientation by examining their association with learners’ COS in a competitive gamified learning environment. To this end, we first analyzed the effects of congruence between learners’ COS and competitive game elements on psychological factors and task performance. Subsequently, we investigated the association between COS and ERP amplitudes to assess the validity of objective assessments of competitive orientation. This study secondarily aimed to assess whether ERP features contribute to the prediction of individual COS. We constructed an SVR model using multimodal features, including ERP features, task performance, and covariates, to examine the contribution of ERP features to the prediction of COS.

Regarding the first research objective, we assessed the association of the congruence between COS and competitive elements in gamified learning with psychological factors related to task motivation and task performance. The results demonstrated that the association between COS and motivation depended on the task goal. Specifically, for learners with low COS, competitive goal framing was associated with lower motivation than self-referential goal framing. This finding aligns with prior research in gamification and achievement motivation showing that competitive elements can undermine motivation for individuals with a low competitive orientation^16^. These findings highlight the importance of considering individual differences among learners and designing competitive elements flexibly according to competitive orientation, rather than implementing them uniformly for all learners.

Interestingly, rather than showing an incongruence effect, task scores were higher in the self-referential condition, suggesting that task performance was influenced by the structure of the task goals (self-referential or competitive) rather than by individual differences in competitive orientation. According to goal-setting theory^33^, clearly defined goals improve performance by specifying what needs to be achieved. This may explain the superior performance observed in the self-referential condition, which emphasized a clearly defined goal score rather than relative performance against an opponent. However, the present design does not allow a clear distinction between the effects of goal reference (self-referential vs. competitive) and goal specificity (clearly defined vs. relative goals), an issue that warrants further investigation. Notably, even in the absence of immediate congruence or incongruence effects on task performance, previous research has identified psychological factors, including motivation, as predictors of dropout in online learning environments^34^. Thus, reducing incongruence between learners’ competitive orientation and competitive goals can mitigate declines in motivation and may encourage long-term academic attainment. Overall, these results indicate that aligning gamified learning designs with individual differences by matching learners’ competitive orientations can prevent declines in psychological factors associated with task motivation, which may be valuable for promoting sustainable learning.

The analysis revealed that the response of the RewP varied according to whether the goal structure was self-referential or competitive, and that its association with COS also differed between conditions. Specifically, a significant negative association between RewP amplitude and COS was observed under the competitive condition, whereas no such association was observed under the self-referential condition. In addition, simple effects analyses indicated that RewP amplitudes were reduced in the competitive condition relative to the self-referential condition at high levels of COS. One interpretation of these findings is that learners with high COS may place greater subjective value on outperforming others, which in turn inflates their expectations for successful outcomes under competitive goals. The RewP reflects reward prediction error elicited by the outcomes of one’s actions^28,29^. Elevated expectations diminish the magnitude of positive prediction errors following correct responses, resulting in attenuated RewP amplitudes. However, heightened success expectancy could, in principle, also influence responses to negative outcomes by increasing the prediction error associated with negative events. Another interpretation is that individuals with high COS, who prioritized “winning the competition” as their ultimate reward, may have assigned lower subjective reward value to trial-level positive feedback indicating correct responses. Previous studies have demonstrated that the RewP also reflects neural responses to rewarding stimuli and is modulated by reward sensitivity; thus, reductions in RewP amplitude are interpreted as reflecting diminished reward sensitivity^35,36^. In the competitive condition, individuals with high COS may have regarded the final competition outcome, which remained unknown until the end of the session, as their primary reward. As a result, the subjective reward value of each instance of positive feedback during task performance may have been reduced, leading to attenuated RewP amplitudes. Because this effect primarily relates to reward valuation of positive feedback indicating correct responses, the FRN, which is elicited by negative feedback, may not have been affected. However, this interpretation was not directly assessed in the present study. Therefore, future studies employing larger samples will be necessary to further elucidate the cognitive and neural mechanisms underlying the attenuation of RewP amplitude in individuals with high competitive orientation under competitive goal structures. A further possible interpretation is that participants with high COS experienced a decline in perceived chances of success following early errors within a session, which in turn led to reduced motivation and attenuated RewP responses. Although this interpretation is theoretically plausible, the present results do not provide evidence that within-session motivational changes account for the observed association between COS and RewP. To examine potential temporal effects within a session, we conducted an additional LME analysis that included a continuous trial index variable representing trial order, as well as its interactions with COS and condition. No significant effects involving the trial index were observed, suggesting that the association between COS and RewP cannot be explained by progressive motivational decline within a session. Details of this analysis are reported in Supplementary Table 3.

Furthermore, this study revealed a negative association between P300 amplitude in response to incorrect feedback and COS, regardless of goal condition. P300 amplitude popularly increases when greater attentional resources are allocated to stimuli^25^. Previous studies have reported a positive association between P300 in response to incorrect feedback and subjective motivation^24^. In the previous study, participants were instructed to achieve predefined goal scores in a mental arithmetic task. Within such a goal structure, negative feedback signaling incorrect responses constituted task-relevant information that could directly contribute to performance improvement. Accordingly, motivation to attain the goal scores was interpreted as being associated with increased attentional allocation to negative feedback, as reflected by larger P300 amplitudes. Based on this line of reasoning, the present finding might initially be interpreted as indicating reduced task motivation among individuals with high COS. However, rather than necessarily reflecting diminished motivation, this pattern may instead suggest differences in attentional allocation depending on competitive orientation. Specifically, individuals with high COS may focus on outcomes of whether the goal is attained rather than on the outcome of each individual problem. As a result, each instance of negative feedback may receive comparatively fewer attentional resources, allowing individuals to disengage more rapidly from negative information and refocus on subsequent trials in pursuit of the overarching goal. Notably, the reduction in P300 amplitude was observed regardless of condition. One possible explanation is that individuals with high COS may have construed even self-referential goals, such as achieving a personal best, within an inherently competitive framework, effectively engaging in competition with themselves. Consequently, similar attentional allocation strategies toward negative feedback may have emerged regardless of whether the goal structure was self-referential or competitive. Regardless of this interpretation, FRN amplitudes did not show a significant association with COS, which may suggest a dissociation between the FRN and the P300. The previous research suggests that the FRN reflects an early, semi-automatic stage of outcome evaluation, whereas the P300 is sensitive to later evaluative processes that depend on the allocation of attentional resources^37^. The absence of corresponding effects in the FRN suggests that early stages of outcome evaluation were not detectably influenced by competitive orientation, whereas later, attention-dependent processing was modulated. However, we cannot rule out the possibility that the limited sample size reduced the statistical power to detect FRN effects. Moreover, because this dissociation was not directly tested, this interpretation remains speculative. In contrast, P300 amplitudes increased across sessions in the competitive condition, indicating that repeated exposure to competitive goals may enhance attentional engagement with negative feedback over time. Unlike self-referential goals, which tend to adjust gradually with improvements in personal performance, competitive goals are inherently relational and remain uncertain until the end of the session. Consequently, negative feedback in competitive contexts may progressively gain psychological significance, as each loss directly affects one’s relative competence, whereas failure to surpass one’s previous best does not necessarily negate self-evaluations of competence. Moreover, in competitive contexts, competence relative to an opponent becomes more concrete as the session progresses, heightening the perceived impact of each loss and potentially accumulating psychological pressure. In sum, this study reveals that COS is associated with a reduction in the RewP to positive outcome feedback indicating a correct response during the pursuit of a competitive goal, as well as a reduction in the P300 to negative outcome feedback indicating an incorrect response, regardless of whether the goal is competitive or self-referential. Future studies with larger sample sizes will be necessary to test the validity of these interpretations regarding the role of individual differences in competitive orientation in modulating outcome evaluation processes during competitive goal pursuit.

The second objective of the present study was to examine whether ERP features contribute to the prediction of COS. An SVR model, which was trained on multimodal features—including ERP features collected under the competitive condition, as well as behavioral and covariate features—showed a moderate but significant correlation between the actual and predicted COS values (*r* = 0.468). Although other features, including the FRN, also contributed to the predictive model, the RewP emerged as the most informative ERP component overall. These findings suggest that ERP components—particularly the RewP—can provide useful information for estimating individual differences in competitive orientation when combined with behavioral measures. To further clarify the role of ERP features, we additionally conducted a regression analysis using the most informative ERP components, FRN and RewP. Although the predictive performance of this reduced model was lower than that of the full multimodal model, this analysis helps delineate the contribution of ERP features and underscores the benefit of integrating behavioral and contextual information. Self-report methods have been widely used to estimate individual differences relevant to game design^19^. However, as noted in the Introduction, self-report measures are affected by various response biases^21^, which can lead to inaccurate assessments of learners’ competitive orientation. This study demonstrates that EEG-based assessments during the learning experience can provide objective information relevant to estimating individual differences in competitive orientation, thereby complementing traditional self-report measures. A previous study^38^ investigated the use of physiological signals to adapt computer games; in this context, EEG provides a distinct advantage due to its high temporal resolution, which enables the capture of cognitive changes as they unfold during task performance. This makes EEG a promising foundation for developing personalized gamification systems. The prediction performance of the regression model in this study was moderate, but there is considerable room for improvement. Increasing the size of the training dataset and incorporating more advanced machine-learning techniques may further enhance predictive accuracy. In particular, recent developments have introduced convolutional neural network-based^39^ and Transformer-based algorithms^40^ capable of decoding ERP waveforms. Applying such models, combined with larger and more diverse datasets, could significantly improve COS prediction performance. At the same time, improving prediction performance will require explicitly addressing the contextual sensitivity of ERP components. ERP responses are influenced by factors such as outcome expectancy, recent performance history, and individual traits (e.g., negativity bias). Accounting for variability arising from these factors may enhance the robustness and generalizability of prediction models. In sum, this research suggests the potential of EEG-based assessments to support the estimation of individual differences in competitive orientation, thereby providing useful insights for neuroscience-informed personalized gamification design approaches in which game design elements are adjusted based on individual differences such as competitive orientation.

A limitation of this study is the relatively small sample size, which may limit statistical power and generalizability, including the possibility that the sample does not fully represent the broader population of interest. In addition, the present study did not examine the association between self-referential goals and orientation toward improvement and learning, which is regarded as a core aspect of mastery-approach goals within achievement goal theory^17^. Therefore, future research should examine whether ERP components are associated with mastery goal orientation during the pursuit of improvement and learning. Examining this relationship would provide a more comprehensive understanding of how cognitive processes involved in outcome evaluation during learning relate to learners’ goal orientations.

In conclusion, this study investigated neurophysiological associations between competitive goal structures in gamified digital learning environments and individual differences in competitive orientation. The findings indicate that motivation toward goal attainment differed depending on the congruence between COS and competitive goal structures, with motivation being undermined under conditions of incongruence. Moreover, ERP responses associated with outcome evaluation processes—particularly the RewP in the competitive condition and the P300 regardless of goal condition—were associated with COS. In addition, exploratory predictive analyses using SVR suggested that multimodal features combining ERP responses and behavioral data collected under competitive goal structures contained predictable information relevant to estimating learners’ competitive orientation, with the RewP contributing most strongly to the prediction. This, in turn, may offer insights relevant to the future development of personalized gamification approaches that take learners’ competitive orientation into account. These findings indicate the feasibility of using neurophysiological responses during learning to estimate individual differences in competitive orientation. Ultimately, this line of research may pave the way for personalizing game elements according to such differences, thereby informing the development of neuroscience-based educational support systems.

## Methods

### Participants

The study included 28 healthy adults (12 females and 16 males; mean age = 26.86 years, standard deviation = 6.38, age range = 20–39 years). All participants had normal or corrected-to-normal vision and reported no history of psychiatric, attention deficit, or neurological disorders. Before data collection, the experimental procedures were explained, and all participants provided informed consent. To create a competitive situation, participants were recruited through a recruitment agency and paired based on predefined conditions and availability. These conditions required participants to be of the same sex, have an age difference of no more than five years, and be unfamiliar with each other. Mathematical ability was not considered in the pairing process; however, as described later, task difficulty was individually adjusted for each participant without their awareness. The paired participants met face-to-face for the first time immediately before the experiment began. The Ethics Committee for Human Research of National Institute of Information and Communications Technology approved the study. The study was carried out in accordance with the Declaration of Helsinki.

### Gamified mental arithmetic task

The gamified mental arithmetic tasks were performed in individual experimental booths within a dimly lit and soundproof room. Participants performed the task in individual experimental booths equipped only with a display, mouse, and numeric keypad, and they could not see their opponent’s screen or the stimulus presentation computer. This design ensured identical experimental environments across the conditions. The competitive and self-referential conditions shared identical task structures and game elements; the only differences between conditions were the goal definition and whether the goal score was displayed before the session. Participants were seated in comfortable chairs and interacted with a custom GUI application developed in MATLAB (MathWorks, Inc., USA), displayed on a monitor positioned in front of them. The task required participants to solve a series of addition problems and input their answers. Game elements included a scoring system according to task performance and a 12-s time limit imposed on each addition problem, with bonus points awarded based on response time. Participants received 2 points for each correct answer and an additional 1 point if they answered within 9 s (75% of the time limit).

**Fig. 7:**
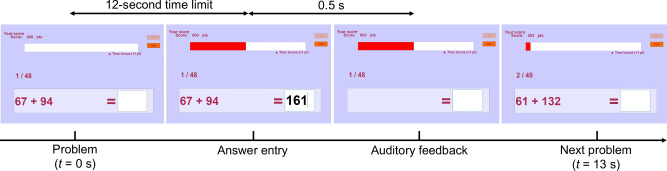
Procedure for each addition problem in the gamified mental arithmetic task. Each trial began with the presentation of an addition problem accompanied by a progress bar indicating the elapsed time, and participants entered their answers using a numeric keypad within a 12-s time limit; auditory feedback was delivered through earphones 500 ms after answer entry, and the total duration of each trial was fixed at 13 s.

Figure 7 illustrates the procedure for each addition problem. Participants initiated the session by clicking the start button with the mouse. As the first addition problem appeared, a progress bar displayed the elapsed time for that addition problem. Answers were entered using the numeric keypad. Auditory feedback was provided through earphones 500 ms after submitting the answer, indicating whether the response was correct (ding sound) or incorrect (buzzer sound). A 500-ms interval was introduced between answer entry and feedback to prevent motion artifacts from contaminating the ERP baseline time window. Incorrect feedback was automatically given if a participant failed to respond within the time limit. To ensure consistency in completion time across participants, the duration of each addition problem was fixed at 13 s, regardless of when the answer was entered.

### Procedure

**Fig. 8:**
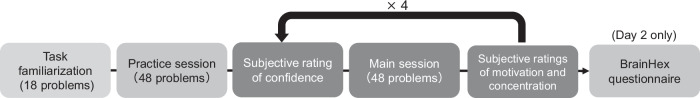
Experimental procedure for each day. On Day 1, participants completed 18 addition problems to familiarize themselves with the task, followed by a practice session (48 problems), and then performed four main sessions (48 problems each), providing a subjective rating of confidence before each session and subjective ratings of motivation and concentration after each session. On Day 2, the procedure was identical to Day 1, and participants additionally completed the BrainHex questionnaire following the final session.

Tasks for each condition were conducted with the same participant pairs over 2 days (days 1 and 2), with no more than 1 week between them. The experimental flow for each day was as follows (Fig. 8). Participants first completed 18 addition problems to familiarize themselves with the task, followed by a practice session with 48 addition problems across six difficulty levels (eight addition problems per level). This session was used to calibrate the task difficulty for each participant. Difficulty levels were identified based on the number of additions (ranging from 1 at level 1 to 3 at level 6) and the number of carry-over operations required (from 0 at level 1 to 3 at level 6). For instance, “74 + 28” (level 2) involved one addition and one carry operation, whereas “74 + 28 + 59 + 35” (level 5) involved three additions, two with carry. Participants were instructed to add numbers from left to right to ensure that the number of carry operations remained constant regardless of the calculation order. The percentage of correct answers was calculated for three consecutive difficulty levels, and the levels closest to 70% accuracy were utilized in the main sessions. However, to prevent changes in task difficulty between conditions, a practice session was conducted on the second day, but the difficulty level was set to be the same as on the first day. Participants were unaware that the difficulty level of the addition problems was tailored to each participant, although the specific addition problems may have differed across participants.

After the practice session, participants completed four main sessions in either condition with each session consisting of 48 addition problems categorized into three difficulty levels (16 addition problems per level), which were identified in the practice session. In the self-referential condition, participants were instructed to surpass their highest score so far, which was displayed on the monitor before each session. Participants were tested in pairs in both the competitive and self-referential conditions to ensure identical experimental settings across conditions, including social presence. Importantly, in both conditions, participants performed the task in separate experimental booths and were unable to communicate with each other. Participants received no information about their opponent’s performance. Thus, in the self-referential condition, the experimental design did not introduce competitive elements into the task.

In the competitive condition, participants were instructed to outperform the score of their partner. Participants were not provided with their opponent’s score in real time. To equate the criterion for goal achievement across conditions, goal achievement was determined according to whether participants exceeded their best score so far, regardless of the condition. This information was withheld from participants. This adaptive criterion for each participant was designed not only to ensure comparable opportunities for goal achievement across participants and conditions, but also to reduce excessive discouragement resulting from repeated failure to achieve goals and to maintain task engagement throughout the experiment by preventing goals from becoming overly difficult. The goal score for the first main session was set at twice the practice score. During the practice sessions, participants solved eight addition problems at each of six difficulty levels. In contrast, the main sessions used only three selected difficulty levels, with 16 problems per level. Hence, for the three difficulty levels used in the main session, participants had practiced with only half the number of problems. Therefore, the practice score was doubled to set a comparable goal for the main session. After each session, visual feedback (“failure” or “success”) was provided to indicate whether the goal had been achieved. Importantly, although the feedback format was identical across conditions to maintain identical experimental environments, its interpretation differed by condition: in the competitive condition, participants were instructed that success depended on outperforming their opponent, whereas in the self-referential condition, success depended on exceeding one’s own best score. The order of experimental conditions was counterbalanced across pairs.

Participants rated their subjective confidence, motivation, and concentration using single-item measures on a 7-point Likert scale (1 = strongly disagree, 7 = strongly agree). Before each session, confidence was assessed using a condition-specific item: in the self-referential condition, “Do you feel confident that you can exceed your personal best score?”; in the competitive condition, “Do you feel confident that you can outperform your opponent’s score?”. After each session, motivation was assessed using a condition-specific item: in the self-referential condition, “How strong was your motivation to exceed your personal best score?”; in the competitive condition, “How strong was your motivation to outperform your opponent’s score?”. Concentration was assessed after each session using the same item in both conditions: “Were you able to concentrate your attention and effort on the task?”. On Day 2, after the main sessions, participants’ competitive orientation was assessed with the BrainHex questionnaire^19^, which classifies players into seven personality types: seekers, survivors, daredevils, masterminds, conquerors, socializers, and achievers. The questionnaire, professionally translated into Japanese and verified by the authors, included a five-point rating of video game experience preferences and a ranking of seven gaming experiences. A score was calculated for each type based on questionnaire responses, and the conqueror score—representing competitive orientation—was used as the COS. The COS is a unidimensional continuous index derived from multiple questionnaire items and ranges from − 10 to 20, with higher scores indicating a stronger competitive orientation.

### EEG Data Measurements

EEG data were recorded from Fpz, Fz, Cz, Pz, and Oz locations based on the International 10–10 system utilizing a wireless portable device (Polymate Mini AP108, Miyuki Giken Co., Ltd., Japan). These locations were selected to cover scalp regions associated with FRN (fronto-central), RewP (fronto-central), and P300 (parietal) distributions. To correct ocular artifacts, horizontal and vertical electrooculograms (EOGs) were recorded from the outer canthi and above the left eye, respectively. The ground and reference electrodes were placed on the left and right mastoids, respectively. EEG measurements at Fz, Cz, Pz, and Oz were obtained using active dry electrodes, whereas adhesive gel disposable electrodes were utilized for Fpz, reference, ground, and EOG recordings.

### EEG Data Preprocessing

Data analysis excluded one participant due to a lack of data for the self-referential condition, caused by an error in the experimental procedure, and absence during the data collection for the competitive condition. Consequently, data of the paired participant in the competitive condition was also removed from the analysis. Further, data from two participants in the competitive condition were not recorded due to procedural errors. The EEG analysis excluded four sessions of the competitive condition and three sessions of the self-referential condition due to recording trouble.

The raw EEG data were preprocessed with the FieldTrip toolbox^41^ and EEGLAB^42^ for MATLAB. Preprocessing was performed per participant and session. The raw EEG and EOG signals were first band-pass filtered between 1 and 50 Hz (− 6 dB cut-off frequencies) using a zero-phase, Kaiser-windowed finite impulse response (FIR) filter (filter order = 908) to stabilize subsequent artifact correction by attenuating slow drifts and high-frequency noise, including line noise. To suppress transient, large-amplitude artifacts, the artifact subspace reconstruction was then applied^43,44^. Ocular artifacts were corrected using a regression-based approach. EEG signals were regressed onto EOG channels together with a constant term. The regression coefficients corresponding to the EOG channels were then used to reconstruct the EOG-related component of the EEG, which was subsequently subtracted from the original EEG signals, while the constant term was retained. The effectiveness of this procedure was confirmed by a reduction in EEG-EOG correlations after correction. Before correction, the mean absolute EEG-EOG correlation, averaged across conditions, sessions, and electrodes, was 0.371. After correction, this value decreased to 6.54 × 10^−16^. To restrict the signal to the frequency range relevant for ERP analyses and to further improve the signal-to-noise ratio prior to single-trial ERP quantification, a zero-phase, Kaiser-windowed FIR low-pass filter was applied at 20 Hz (− 6 dB cut-off frequency, filter order = 364). Continuous EEG data were segmented from − 200 ms to 600 ms, relative to the onset time of each auditory feedback. In the analysis, the response to the feedback of each answer was considered one trial. Baseline correction was performed using the mean amplitude of the pre-stimulus region. FRN and RewP are distributed in a frontocentral region and P300 in a parietal region on the scalp; thus, data from the Fz and Pz electrodes were included for further analysis^24^. Trials contaminated by artifacts were identified using a threshold of ± 85 μV, and those exceeding this threshold were eliminated from further analysis. The ERP analysis excluded timed-out trials^24^ because the return of negative feedback in these cases was predetermined and could have caused different outcome processing.

Single-trial mean amplitudes of the FRN and RewP at Fz and the P300 at Pz were calculated for each EEG trial to enable trial-level modeling using LME analyses, which allowed ERP responses to be directly linked to trial-specific covariates (e.g., difficulty level and local frequency). The FRN/P300 and RewP were calculated using incorrect and correct trials, respectively. The mean number of trials included in the ERP analyses across participants was as follows (mean ± SD): for the self-referential condition, 151.0 ± 26.4 correct trials and 25.5 ± 13.9 incorrect trials; for the competitive condition, 147.0 ± 38.5 correct trials and 23.0 ± 11.5 incorrect trials. The time windows for calculating the mean amplitudes were identified based on the grand-averaged ERP waveforms, including trials from both conditions: 50 ms for FRN, 75 ms for RewP, and 90 ms for P300, centered on the peak latency of each grand-averaged ERP. The widths of the time windows were determined by visual inspection of the grand-averaged ERP waveforms.

### Statistical analysis

Our hypothesis is based on the idea that congruence between an individual’s COS and competitive game elements improves psychological factors, such as task motivation and engagement toward goal attainment, whereas a mismatch may worsen them. ERP components, such as P300, FRN, and RewP, reflect changes in attentional allocation and reward prediction error processes during goal pursuit—both of which are influenced by psychological attitudes toward goal attainment—variations in ERP amplitudes may capture this match or mismatch. Therefore, ERPs may contribute to estimating competitive orientation, providing a means to tailor gamification elements according to the orientation.

To investigate this hypothesis, the statistical analyses were structured in two parts. First, we assessed the foundational assumption that congruence (or incongruence) between COS and competitive game elements affects psychological factors. Specifically, we analyzed the effects of COS, condition (i.e., competitive vs. self-referential), and their interaction on subjective ratings of motivation, confidence, and concentration. Considering that these psychological factors influence academic achievement^45^, we investigated their effects on task performance, i.e., task scores. Second, we assessed the association of ERP with individual differences in competitive orientation in competitive contexts. In this analysis, we hypothesized that the association between the mean amplitudes of ERP components and COS would differ based on the condition—that is, a significant association would be observed under the competitive condition, rather than under the self-referential condition.

To investigate the effects of congruence between COS and competitive game elements on psychological factors and task performance (i.e., task score), we used an LME. To analyze subjective ratings, the model included COS (continuous), condition (categorical: competitive or self-referential), and their interaction as well as the session (continuous) and its interaction with the condition as fixed effects. The session was included to account for potential temporal changes in participants’ responses as the task progressed. In particular, in the self-referential condition, the target score gradually increased over sessions and was explicitly communicated to participants. The interaction between session and condition was included to capture this potential modification. Task score (continuous) was added as a covariate in the subjective rating analysis to control for its potential influence of task performance on participants’ subjective responses. To analyze task performance, the response variable was the task score in each session. All models included random intercepts for participants. In Wilkinson notation, the model for subjective ratings was specified as ${\rm{Subjective}\;{\rm{rating}}} \sim {\rm{COS}}* {\rm{Condition}}+{\rm{Session}}* {\rm{Condition}}+{\rm{Task}\,{\rm{score}}}+(1| {\rm{Participant}})$ and for task performance: ${\rm{Task}\,{\rm{score}}} \sim {\rm{COS}}* {\rm{Condition}+{\rm{Session}}}* {\rm{Condition}}+(1| {\rm{Participant}})$.

Sum-to-zero contrasts were applied to categorical predictors, and continuous fixed-effect predictors were standardized before analysis. The significance of fixed-effect coefficients was assessed using type III *F*-tests with the Satterthwaite approximation. Post hoc analyses followed significant interactions. We examined the significance of the simple slopes of COS within each condition for significant interaction of COS by condition. In addition, we examined the simple effects of condition by comparing the competitive and self-referential conditions at low (− 1 SD), mean, and high (+ 1 SD) levels of COS. P values were adjusted for multiple comparisons using the Holm method. All analyses were conducted in R using the afex^46^ and emmeans^47^ packages for model estimation and post hoc testing. The significance level was set at *α* of 0.05.

To investigate the effects of congruence between COS and competitive game elements on ERP components, we applied LME models separately for each ERP component. The single-trial mean amplitudes of RewP, FRN, and P300 were used as response variables in a single trial analysis approach^32^. The model included COS, condition, and their interaction, as well as session and its interaction with the condition, as fixed effects. In addition, several covariates were added to control for known effects on ERP amplitudes. First, mental effort during arithmetic problem-solving affects these ERPs^48,49^; thus, the difficulty level of each addition problem (difficulty; treated as a continuous variable ranging from Level 1 as easiest to Level 6 as hardest) was included as a covariate. Second, the probability of a target stimulus affects the P300 component^50^, and both the FRN and RewP reflect the prediction error between expected and actual outcomes^51^; thus, these components are sensitive to expectancy. Therefore, expectancy-related effects were modeled using a variable termed local frequency. This was defined as the number of consecutive incorrect trials preceding a correct response for RewP and the number of consecutive correct trials preceding an incorrect response for FRN and P300. The model for ERP analysis in Wilkinson notation was specified as ${\rm{Amplitude}} \sim {\rm{COS}}* {\rm{Condition}}+{\rm{Session}}* {\rm{Condition}}+{\rm{Difficulty}}+{\rm{Local}\; {\rm{frequency}}}+(1| {\rm{Participant}})$.

A session that did not include any incorrect trials was excluded from the analysis of FRN and P300. This criterion affected 10 participants and resulted in the exclusion of 19 sessions (8.8% of all sessions). Importantly, all affected participants contributed data from at least one remaining session. All other analytical procedures, including contrast coding, continuous variable standardization, and post hoc testing, were identical to those described for the subjective rating analyses.

### Prediction of COS

The second objective was to examine whether ERP features contribute to the prediction of competitive orientation when executing gamified learning materials with competitive elements. To this end, an SVR model with a linear kernel was trained using LOOCV, in which data from one participant were utilized as the test set while using the remaining participants’ data for training. This process was repeated until each participant had been used once as the test case.

The features used for prediction included ERP measures (mean amplitudes of RewP to correct trials, FRN to incorrect trials, and P300 to incorrect trials), as well as task scores. We used data from the competitive condition, as this analysis aimed to investigate the prediction ability of ERP features on individual differences in COS, specifically in competitive contexts, where the congruence or incongruence between competitive orientation and task goals is most salient. To account for potential confounding factors that may affect ERP amplitudes, covariates used in the LME analysis were included a priori in the predictive models. Local frequency based on correct trials was not included because it conveys largely overlapping information with the incorrect-trial-based measure and would increase redundancy in the feature set. ERP responses, the difficulty level of incorrect trials, the difficulty level of correct trials, and the local frequency of incorrect trials were originally defined at the single-trial level. Therefore, these features were first averaged within each session and then averaged across all sessions for each participant. In contrast, task score represents a session-level outcome and was averaged across sessions. Hence, the final feature set comprised seven dimensions, consisting of 3 ERP features, 1 task performance feature (score), and 3 covariate features. Three participants who lacked data in the competitive condition were excluded, resulting in a final sample size of 24.

During model training, each feature was standardized according to statistics computed from the training data. The SVR hyperparameters—cost and epsilon—were optimized using a grid search with threefold cross-validation on the training set, assessed using the MAE. The parameter ranges were cost = [1, 10, 100], epsilon = [0.05, 0.1, 0.15]. Model performance was assessed using the Pearson correlation coefficient (*r*) and the MAE between predicted and actual COS values. To examine the contribution of each feature to the prediction, the mean absolute values of the regression coefficients assigned to each feature across all LOOCV-trained models were interpreted as feature importance scores.

In addition, we evaluated other regression models, including ridge regression, lasso regression, and elastic net regression, using the same feature set and cross-validation procedure. For ridge regression, the regularization parameter *α* was optimized over the range [0.01, 0.1, 1]. For lasso regression, *α* was optimized over [0.001, 0.01, 0.1]. For elastic net regression, *α* was optimized over [0.001, 0.01, 0.1], and the mixing parameter l1 ratio was optimized over [0.2, 0.5, 0.8]. The regression analysis was conducted using Python (version 3.11.9) and the scikit-learn library (version 1.5.2)^52^.

## Supplementary information

Supplementary information

## Data Availability

The dataset used in this study is available from the corresponding author upon reasonable request. Only data for which participants have provided consent for public sharing can be made available.

## Code availability

The code used in this study is not publicly available but is available from the corresponding author upon reasonable request to qualified researchers.
