# Supplementary material for: Neurophysiological prediction of competitive orientation via EEG during outcome evaluation in gamified learning
Source: NPJ Sci Learn. 2026 Apr 6;11:34. doi: 10.1038/s41539-026-00420-y (PMC13230860; doi:10.1038/s41539-026-00420-y)
Supplement: Supplementary file 1 — Supplementary information [file 41539_2026_420_MOESM1_ESM.pdf]

Supplementary Tables

Supplementary Table 1. Predictive performance of alternative regression models.

Model	MAE	Pearson's r	p-value
Linear SVR	3.68	0.468	0.021
Ridge	3.65	0.322	0.125
Lasso	3.62	0.286	0.175
Elastic Net	3.58	0.296	0.160

Notes. p-values correspond to Pearson's correlation tests.

Supplementary Table 2. Predictive performance of the regression model using a reduced feature set that included the only two ERP features (RewP and FRN)

Feature set	MAE	Pearson's r	p-value
RewP + FRN	3.64	0.346	0.098

Notes. p-values correspond to Pearson's correlation tests.

Supplementary Table 3. Results of linear mixed-effects models examining the effects of trial index on Reward Positivity (RewP) amplitudes.

Model:
$\text{RewP} \sim \text{COS} * \text{Condition} * \text{Trial index} + \text{Session} * \text{Condition} + \text{Difficulty} + \text{Local frequency} + (1|\text{Subject})$

Fixed effects	Estimate (β)	SE	F	p	Significance
Intercept	3.79	0.32	–	–	–
COS	–0.63	0.33	3.63	0.068	–
Condition	0.13	0.07	3.93	0.048	*
Trial index	–0.11	0.07	2.63	0.105	–
Session	0.26	0.07	15.62	$< .001$	*
(Difficulty)	0.57	0.11	29.54	$< .001$	*
(Local frequency)	0.06	0.07	0.64	0.423	–
COS × Condition	0.13	0.07	4.06	0.044	*
COS × Trial index	–0.02	0.07	0.14	0.707	–
Condition × Trial index	0.02	0.07	0.06	0.812	–
Condition × Session	–0.06	0.07	0.78	0.376	–
COS × Condition × Trial index	0.09	0.07	1.84	0.175	–

Notes. SE = standard error. * $p < 0.05$. Covariates are shown in parentheses.
